# Symptoms from Morphine Sulphate

**Published:** 1896-12

**Authors:** E. V. Ross

**Affiliations:** Rochester, N. Y.


### SYMPTOMS FROM MORPHINE SULPHATE.

E. V. Ross, M. D., Rochester, N. Y.

A lady, æt. thirty, dark complexioned, took one-eighth grain of Morphine Sulphate for a very painful dysmenorrhœa. The following symptoms were produced:

*Great anxiety, thought she was going to die.*

Vertigo, objects seemed to be turning in a circle.

*Head felt as heavy as lead, could scarcely hold it up.*

Pupils contracted.

Delusion of vision on closing eyes, sees a man standing at foot of bed. The room seems full of white and colored babies.

Nose.—*Intense itching, tingling, numb feeling on end of nose, rubs it constantly.*

Nausea with repeated attacks of vomiting.

Violent palpitation with throbbing of carotid.

Heaviness and weight of lower extremities.

Weakness and trembling of lower extremities. Felt very weak.

Numb feeling all over.

Spells of feeling faint, come on suddenly, with great anxiety; thought she was going to die.

Intolerable itching of skin.

These symptoms lasted twenty-four hours and gradually subsided.

[See "Symptoms from Morphine," by Dr. R. L. Thurston, in the December, 1895, number, page 563; also, "Confirmation of Morphine Symptoms," by Dr. Thomas Skinner, March number, page 123; also, "Vertigo Worse from Motion," by Dr. C. M. Boger, June number, page 295.—EDITOR.]

